# Books and Authors

**Published:** 1909-08

**Authors:** 


### BOOKS AND AUTHORS.

**Progressive Medicine.** A Quarterly Digest of Advances, Discoveries and Improvements in the Medical and Surgical Sciences. Edited by **Hobart Amory Hare,** M.D., Professor of Therapeutics and Materia Medica in the Jefferson Medical College of Philadelphia. Vol. XI. No. 2, June, 1909. Octavo, 317 pages, with 52 illustrations. Per annum, in four paper-bound volumes. Lea & Febiger, Publishers, Philadelphia and New York. ($6.00 in cloth, $9.00, net prices.)

The contents of this number comprise hernia, by William B. Coley; surgery of the abdomen, by Edward Milton Foote; gynecology, by John G. Clark; diseases of the blood, diathetic and metabolic diseases; diseases of the spleen, thyroid gland, and lymphatic system, by Alfred Stengel; and ophthalmology, by Edward Jackson. Reference is made in the hernia article to Erdmann's papers in the *Annals of Surgery* (Feb., 1909,) calling attention to certain accidents in hernia operations, in which he refers to Skeel's paper read before the American Association of Obstetricians and Gynecologists, at Baltimore, September, 1908, where the subject was fully discussed.

This entire number is an excellent one. The hernia article is full of interest.

**Textbook of Embryology,** by **Frederick Randolph Bailey,** A.M., M.D., Adjunct Professor of Histology and Embryology, College of Physicians and Surgeons (Medical Department of Columbia University), and **Adam Marion Miller,** A.M., Instructor in Histology and Embryology at the same institution. With 515 illustrations. New York: William Wood & Company. 1909.

The study of embryology is coming to be,—even now is,—recognised as one of the important features of the preparation for a medical degree. Or, perhaps it would be better to say that nowadays no physician can be considered properly equipped without a knowledge of embryology. It has come to be recognised as a part of the foundation of medical study.

The work before us is, without doubt, the largest in scope of any American treatise on this topic. The authors are qualified to give instruction in embryology quite beyond the average teachers of histology, and embryology, and have created a work that challenges criticism. The book is generously illustrated, the pictures being of a high class, conveying clear conception of the structures illustrated.

The practical suggestions, in concise form, added at the end of each chapter are useful hints, especially for work in the laboratory, while the references are most convenient for the further study of the subjects.

The heavy calendered paper on which the book is printed brings out the illustrations in sharp detail, as well as gives character to the printed page. It would be difficult to conceive of a treatise better adapted to the purposes for which this one is intended—namely, a textbook on embryology. It is the outgrowth of the course on this subject of the medical department of Columbia University,—the College of Physicians and Surgeons,—but its scope has been broadened so that it becomes of greater value to the general student of embryology and allied sciences. An appendix contains useful hints concerning the better methods of procuring, handling, and preparing embryological material. The book is well rounded out by this addition, and an excellent index serves to make its pages quickly accessible

---

**Treves's Operative Surgery.** New (3d) Edition. A Manual of Operative Surgery. By **Sir Frederick Treves,** Bart., G.C.V.O., C.B., LL.D., F.R.C.S., Sergeant-Surgeon to H. M. the King, Surgeon-in-Ordinary to H. R. H. the Prince of Wales, Consulting Surgeon to the London Hospital; and **Jonathan Hutchinson,** F.R.C.S., Surgeon to the London Hospital. New (3d) Edition, revised and rewritten. In two octavo volumes. Volume I, 775 pages, with 193 engravings and 17 full-page plates. Lea & Febiger, Publishers, Philadelphia and New York ,1909. Half-morocco, $6.50, net.

Sir Frederick Treves is recognised as the foremost British surgeon of the present day. His operation on King Edward for appendicitis some six years ago and his faithful devotion to his royal patient during a long period afterward, will be remembered as one of his greatest triumphs. It will be recalled that the King had postponed the operation on account of the approaching coronation ceremonies, until the danger line had been reached, hence the task of Sir Frederick was more than ordinarily trying, even putting his physical and mental powers to the severest test. Sir Frederick, as is well known, was not found wanting.

Another incident in Sir Frederick Treves's career deserves mention. It was after a heart to heart talk,—a full and free consultation with Mr. Treves that Dr. Wilfred T. Grenfell, the noted physician-missionary who had already determined to devote his life to helping his fellow-men, decided to throw his lot with the deep sea missionaries. Finding work in the North Sea in competent hands, he turned his attention to the urgent needs of the fisher-folk on this side of the Atlantic, where, in Labrador, he has achieved astonishing results in the face of the greatest difficulties. Here, again, we see the great English surgeon giving counsel to an important life-saving enterprise. So much for the author, now for the book itself.

The second edition of what was a manual but which now assumes a more important role, has been out of print sometime, and the author prevailed upon Mr. Hutchinson to make a complete revision. It now includes the more important details of abdominal surgery elaborated from the primary state in former editions, and many other topics have been enlarged, freshly illustrated, or otherwise improved. Numerous colored plates have been inserted, making clearer the anatomic landmarks of certain important operations. A description of the better methods of performing the various operations has been made, unincumbered by foot notes and other details. The technic is simple, the instruments are few in number but the results are satisfactory.

In this volume the operations on the abdomen have been grouped together. It makes a handsome book, fine paper, gilt top, deckled edges, morocco bound; and will ornament the library in every sense. Every surgeon will make haste to possess it.

---

**The Practical Medicine Series.** Ten volumes. Issued under the general editorial charge of **Gustavus P. Head**, M.D., Professor of Laryngology and Rhinology in the Chicago Post-Graduate Medical School. Vol. I, General Medicine, edited by **Frank Billings**, M.S., M.D. Head of the Medical Department and Dean of the Faculty of Rush Medical College, and **J. H. Salisbury**, M.D., Professor of Medicine Chicago Clinical School. Series 1909. Chicago: The Year Book Publishers. (Price, $1.50; entire series, $10.00.)

Again we find Billings and Salisbury have presented here the practical questions of the year as related to general medicine. Tuberculosis naturally claims a prominent place in this volume; diseases of the heart and lungs also are well considered; general diagnosis, likewise, has a generous consideration. Diseases of the blood, infectious diseases, and diseases of the kidney are also well represented in the book. It is an excellent compilation of the literature of the year on these topics and is valuable for the general practitioner, for whom in particular it is prepared.

---

**Writing the Short-Story.** By **J. Berg Esenwein**, A.M., Lit. D. Editor of Lippincott's Monthly Magazine. Author of How to Attract and Hold an Audience. Cloth, 12mo, 448 pages. Price, $1.25. Published by Hinds, Noble & Eldredge, New York.

Nearly everybody either knows how or wants to know how to write a story long or short. This book chiefly concerns those who would write a short story. It will tell all about it. No brains are required,—just read this book which is a handsome volume of 450 pages, full of sensible and helpful ideas for those who write and, indeed, also for those who would intelligently read the short-story. Its chapters on gathering literary materials, and the structure of the plot, are clear and suggestive, while the treatment of dialogue, characters, titles, and opening and closing the story, must prove valuable to experienced writers as well as to beginners. It is the work of a practised writer, and an editor who has handled many thousands of manuscripts from writers great and small. Therefore his advice on how to prepare manuscript, how to sell the story, and all the practical end of the matter, is quite as valuable as his chapters on the technic of the short-story.

**The Practical Medicine Series.** Ten volumes. Issued under the general editorial charge of **Gustavus P. Head,** M.D., Professor of Laryngology and Rhinology in the Chicago Post-Graduate Medical School. Vol. II, General Surgery, edited by **John B. Murphy,** A.M., M.D., LL.D., Professor of Surgery in the Northwestern University; Attending Surgeon and Chief of Staff, Mercy Hospital. Series 1909. Chicago: The Year Book Publishers. (Price, $2.00; entire series, $10.00.)

The compiler pursues in this book the same general plan as heretofore. To begin with, he pays some attention to anesthesia and analgesia,—important topics. Radiotherapy receives due attention some new instruments are presented, operative technic, with asepsis and antisepsis is dealt with, wound healing with pathological interventions is duly considered, antiferment treatment is applied, together with other methods; then tumors, the bloodvessels, the bones, the various regions, visceral surgery, the spine and the extremities.

Other topics are taken up but we need not enumerate them all. The surgery of the stomach is most interestingly dealt with, many illustrations being introduced which serve to explain the technic. The index is very complete. This book is one of the best of the surgical compilations of the year.

---

**Annual Report of the Surgeon-General of the Public Health and Marine Hospital Service of the United States,** giving the operations of the service, fourth fiscal year, 1908. Washington Government Printing Office. 1909.

This volume though small deals with many interesting questions relating to public health, hygiene and sanitation. The plague in California receives detailed attention. Among 160 cases of human plague there were 78 deaths, and 371 plague-infected rats were disposed of. The campaign against rats and to improve conditions in San Francisco is still continued with unabated vigor. Quarantine measures and the medical inspection of immigrants form interesting topics in this report. Public health officers will find in it much material for study or investigation.

---

**International Clinics.** A Quarterly of Illustrated Clinical Lectures and especially prepared articles on Treatment, Medicine, Surgery, Neurology, Pediatrics, Obstetrics, Gynecology, Orthopedics, Pathology, Dermatology, Ophthalmology, Otology, Rhinology, Laryngology, Hygiene and other topics of interest. Edited by **W. T. Longcope,** M.D., Volume II. Nineteenth series. Philadelphia and London: J. B. Lippincott Co. 1909. (Cloth, $2.00.)

We find in this volume articles on treatment (4); on medicine (4); on surgery (4); on gynecology and obstetrics (2); ophthalmology (1); otology (1); proctology (1); psychiatry (1); and on pathology (2). The book contains many illustrations—colored plates, plates in black, and figures in the text. It is an excellent number, dealing with medical questions pertaining to the present time.

**Report of the United States Commissioner of Education,** for the year ended June 30, 1908. Volume I. Washington Government Printing Office. 1908.

This volume has appeared much in advance of the usual time due, as the commissioner explains, to his desire to hasten the publication of the report and thus make its material available sooner than heretofore. The second volume may be expected before many weeks. This one contains much of interest to educators, and points out what is going on in both home and foreign countries, particularly in the Philippines, Porto Rico, Great Britain and Ireland, France in Central Europe and in Spanish-American countries. The commissioner discusses educational standards interestingly, instructively we may say, stating the case from all viewpoints. The volume will be examined with interest by all persons concerned in education, in its improvement, in its status throughout the civilised world.

